# A Novel Approach to Assess Salt Stress Tolerance in Wheat Using Hyperspectral Imaging

**DOI:** 10.3389/fpls.2018.01182

**Published:** 2018-08-24

**Authors:** Ali Moghimi, Ce Yang, Marisa E. Miller, Shahryar F. Kianian, Peter M. Marchetto

**Affiliations:** ^1^Department of Bioproducts and Biosystems Engineering, University of Minnesota, Minneapolis, MN, United States; ^2^Cereal Disease Laboratory, USDA-ARS, Saint Paul, MN, United States; ^3^Department of Plant Pathology, University of Minnesota, Minneapolis, MN, United States

**Keywords:** Bayesian inference, histogram distance, hyperspectral imaging, image processing, machine learning, plant phenotyping, salt stress, wheat

## Abstract

Salinity stress has significant adverse effects on crop productivity and yield. The primary goal of this study was to quantitatively rank salt tolerance in wheat using hyperspectral imaging. Four wheat lines were assayed in a hydroponic system with control and salt treatments (0 and 200 mM NaCl). Hyperspectral images were captured one day after salt application when there were no visual symptoms. Subsequent to necessary preprocessing tasks, two endmembers, each representing one of the treatment, were identified in each image using successive volume maximization. To simplify image analysis and interpretation, similarity of all pixels to the salt endmember was calculated by a technique proposed in this study, referred to as vector-wise similarity measurement. Using this approach allowed high-dimensional hyperspectral images to be reduced to one-dimensional gray-scale images while retaining all relevant information. Two methods were then utilized to analyze the gray-scale images: minimum difference of pair assignments and Bayesian method. The rankings of both methods were similar and consistent with the expected ranking obtained by conventional phenotyping experiments and historical evidence of salt tolerance. This research highlights the application of machine learning in hyperspectral image analysis for phenotyping of plants in a quantitative, interpretable, and non-invasive manner.

## Introduction

Salinity stress is a major abiotic stress that has significant adverse effects on crop productivity and yield. These negative effects include interference of root function in absorbing water, as well as the prevention of physiological and biochemical processes such as nutrient uptake and assimilation (Carillo et al., 2011). Unfortunately, many regions around the world are facing a rapid increase in soil salinity and sodicity. It is estimated that at least 0.3 million hectares of farmland is becoming unusable annually, and another 20–46 million ha are suffering decreases in production potential each year (FAO and ITPS, 2015). Nevertheless, even with lower yield potential, these salt-affected farmlands must continue to produce crops so the increasing demand for food can be met and food security concerns mitigated. The lack of new productive land threatens food security, thus the productivity of existing marginal lands must improve.

There are numerous potential solutions for mitigating salt stress, including genetic engineering of plants with salt tolerance (Agarwal et al., 2013; Wei et al., 2017) and application of exogenous compounds such as hormones, growth regulators, or nanoparticles (Mbarki et al., 2018). Among the potential solutions, selecting plant varieties with high tolerance to salt stress appears to be one of the most promising approaches in utilizing salt-affected soil for crop production (Ondrasek et al., 2011; Sytar et al., 2017). Although some progress has been made using measurement of photosynthetic parameters as a more sensitive method to screen for salt tolerance (Sun et al., 2016; Kalaji et al., 2018), the standard process of selecting either conventionally–bred or transgenic salt-tolerant crop lines relies on laborious phenotyping to assess tolerance. Despite the emergence of innovative platforms, precise instrumentation, sophisticated sensors, and rapid development of advanced machine learning and deep learning algorithms, phenotyping is still a barrier to variety development. While DNA sequencing and plant genotyping has rapidly evolved, phenotyping still depends on conventional methods which are not as accurate or efficient. In general, these techniques can be time-consuming, destructive, subjective, and costly. In recent years, non-contact sensing technology, in particular imaging, has been extensively deployed as a potential substitute for conventional methods for high-throughput phenotyping of plants. Thanks to the advances in developing sensors with high spatial and spectral resolution, different imaging sensors including visible, fluorescence, thermal, and spectral imaging are available, each tailored for specific applications. Each of these sensing technologies can vary in their application, as well as limitations, in the context of plant phenotyping (Li et al., 2014). Among these techniques, hyperspectral imaging (HSI) is uniquely suited to provide insights into the internal activities of plants, leaf tissue structure, leaf pigments, and water content (Mahlein et al., 2012). HSI also provides the ability to investigate physiological dynamics of plants caused by environmental variables (Wahabzada et al., 2016), and consequently has drawn substantial attention for plant phenotyping (Kuska et al., 2015).

Few research studies have attempted to identify salt stress in plants using hyperspectral reflectance. In a previous study, three potential indicators including blue, yellow, and red edge positions of vegetation reflectance spectrum were calculated to detect four levels of salt stress imposed on Chinese castor bean (Li et al., 2010). The authors claimed that blue and red edge positions shift to the shorter wavelength in response to salt stress and therefore could be used to detect salt stress. However, the pattern of shifting to the shorter wavelength was not consistent across all treatments and hence further research is required. In another paper, the application of HSI to identify plant tolerance to salt stress in a high throughput phenotyping system was reviewed (Sytar et al., 2017). They concluded more efficient and fully automated methods are required to analyze complex hyperspectral images.

To leverage the full potential of HSI, a large high-quality hyperspectral dataset and several preprocessing tasks are necessary (e.g., radiometric calibration, normalization, mixed pixel filtering, etc.). However, there are two major challenges that hamper the application of HSI.

The first major challenge is accounting for the variance caused by the complex interaction between incident light and leaf surfaces due to non-Lambertian reflectance properties. The direction of reflected light is a function of leaf geometry, including leaf angle, and curvature. Several researchers have focused on pre-processing techniques to address the problems related to leaf angle and curvature (Behmann et al., 2015; Makdessi et al., 2017; Wendel and Underwood, 2017). One method to resolve this problem is to generate a high-resolution 3D representation of plants by upfront geometric calibration of the hyperspectral camera (Behmann et al., 2015). However, this proposed method depends on highly intensive processing and is only suitable for close-range imaging.

The second major challenge is analyzing the complex and high-dimensional hyperspectral images in order to extract meaningful features and recognize latent patterns associated with the desired phenotyping trait in a more interpretable manner. To address this issue, machine learning (ML) and deep learning algorithms can be leveraged. Recent reviews of various ML algorithms emphasize the potential of these methods in the context of agriculture and provide guidelines for plant scientists to deploy them (Bauckhage and Kersting, 2013; Singh et al., 2016; Coppens et al., 2017). Singh et al. (2016) reported that ML algorithms are a promising approach to analyze large datasets generated by sophisticated imaging sensors (e.g., hyperspectral cameras) mounted to platforms that can cover large areas. Despite several studies that focus on the application of HSI for plant phenotyping, research is limited in the context of handling, processing, and analyzing hyperspectral images.

This research was motivated by the need to identify salt tolerant wheat lines to mitigate yield losses due to salinity, and to ultimately maintain or improve production on saline soils. The objectives of this study were to (i) rank wheat lines based on their tolerance to salt stress, (ii) assess the difference between the salt tolerance of lines to attain a quantitative ranking rather than a qualitative ranking, and (iii) evaluate the feasibility of precise ranking of wheat lines as early as one day after applying salt treatment. We hypothesized that the spectral response of wheat leaves experiencing salt stress would deviate from the control leaves even one day after adding the stress, and this deviation would be larger for a susceptible line compared to a salt tolerant line. To the best of our knowledge, no previous study has investigated early detection of salt tolerant plant lines using advanced phenotyping tools and approaches. This research proposes a machine learning approach to analyze hyperspectral images of wheat lines to rank their salt stress tolerance in a quantitative, interpretable, and non-destructive manner while reducing cost, time, and labor input.

## Materials and methods

### Sample preparation and conventional phenotyping for salt tolerance screening

To develop analytical methods for analysis of hyperspectral images, four bread wheat (*Triticum aestivum*) lines were selected with varying levels of salt tolerance. The cultivar Kharchia was included as it is historically known to maintain a stable harvest index and yield well in high salt conditions (Schachtman et al., 1992; Munns et al., 2006), and the salt-sensitive cultivar Chinese Spring (CS) was selected as well (Zhang et al., 2016). Two additional “unknown” lines were selected for screening from a set of wheat alloplasmic lines developed in Japan (Tsunewaki et al., 1996, 2002). Alloplasmic lines are created by substitution backcrossing to replace the cytoplasmic genomes of one species (in this case, bread wheat) with those of another (in this case, wild wheat relatives) while maintaining the original nuclear genome background, and have shown promise for improving stress tolerance and other developmental traits (Liberatore et al., 2016). The two alloplasmic lines selected were *Aegilops columnaris* KU11-2 (CS) [abbreviated co(CS) hereafter] and *Ae. speltoides aucheri* KU2201B (CS) [abbreviated sp(CS) hereafter] with the cytoplasmic genome type preceding the nuclear genome background, which in this case is Chinese Spring (CS).

Screening was performed in a hydroponic system in a Conviron growth chamber to ensure uniform conditions. Hydroponic systems are commonly used to screen plants for salt tolerance, including wheat. In all experiments, growth conditions in the Conviron were set at 22°C during light conditions and 18°C during the dark, 16 h photoperiod, 375 μmol m^−2^ s^−1^ light intensity, and 50% relative humidity. Three hydroponic tanks were used per treatment (control treatment: 0 mM NaCl and salt treatment: 200 mM NaCl). Each hydroponic tank contained a grid of 16 Cone-tainers (Ray Leach brand) filled with perlite. Within each tank, there were four genotypes each with four individual replicates (4 cone-tainers x 4 genotypes). For each treatment (salt or control), there were three replicate tanks; hence, there were a total of 48 (3 replications × 4 Cone-tainers × 4 genotypes) Cone-tainers for each treatment. The grid was placed into a tank just large enough to hold the grid, and 20 L of hydroponic solution was used per tank. Genotypes were randomly assigned to positions in each cone-tainer grid using the sample and matrix functions in R (version 3.4.0). Aeration was supplied to each tank with an aquarium pump and two large airstones (at either end of the tank). Lines were transplanted into the tanks, and the lights and aeration were switched on 24 h after transplanting. When leaf 1 emerged, $\frac{1}{4}$ strength Hoagland's solution (PhytoTech H353) was added, and the pH was adjusted to 6.5. When leaf 2 emerged, the Hoagland's was increased to $\frac{1}{2}$ strength in all tanks and CaCl_2_ was added to the tanks destined for salt treatment in a 15:1 molar ratio of NaCl to CaCl_2_. When leaf 4 emerged, salt was added to the salt tanks gradually over 2 days to reach a final concentration of 200 mM. The water level and pH (to 6.5) were adjusted 3 times per week throughout the experiment.

To compare the salt tolerance of four wheat lines, both aerial and root biomass was harvested separately for each individual plant 2 weeks after salt treatment was applied. Plant matter was dried at 60-65°C for 4 days and then weighed. Dry weight data were analyzed in R (version 3.4.0) using ANOVA (car package, version 2.1-5) and linear mixed-effect modeling (nlme package, version 3.1-120). For linear mixed-effect modeling analysis, dry weight was considered as the response in the analysis, salt level and genotypes were considered as fixed effects, and tank number was considered as a random effect. Model results were identical if tank position was considered as a nested-random effect of tank number, thus the results with tank number were used as the only random effect. To compare the response of the alloplasmics to the response of the euplasmic parents when the salt level is changed, the coefficient estimates of the lme model were examined.

### Hyperspectral image acquisition

**Figure 1:**
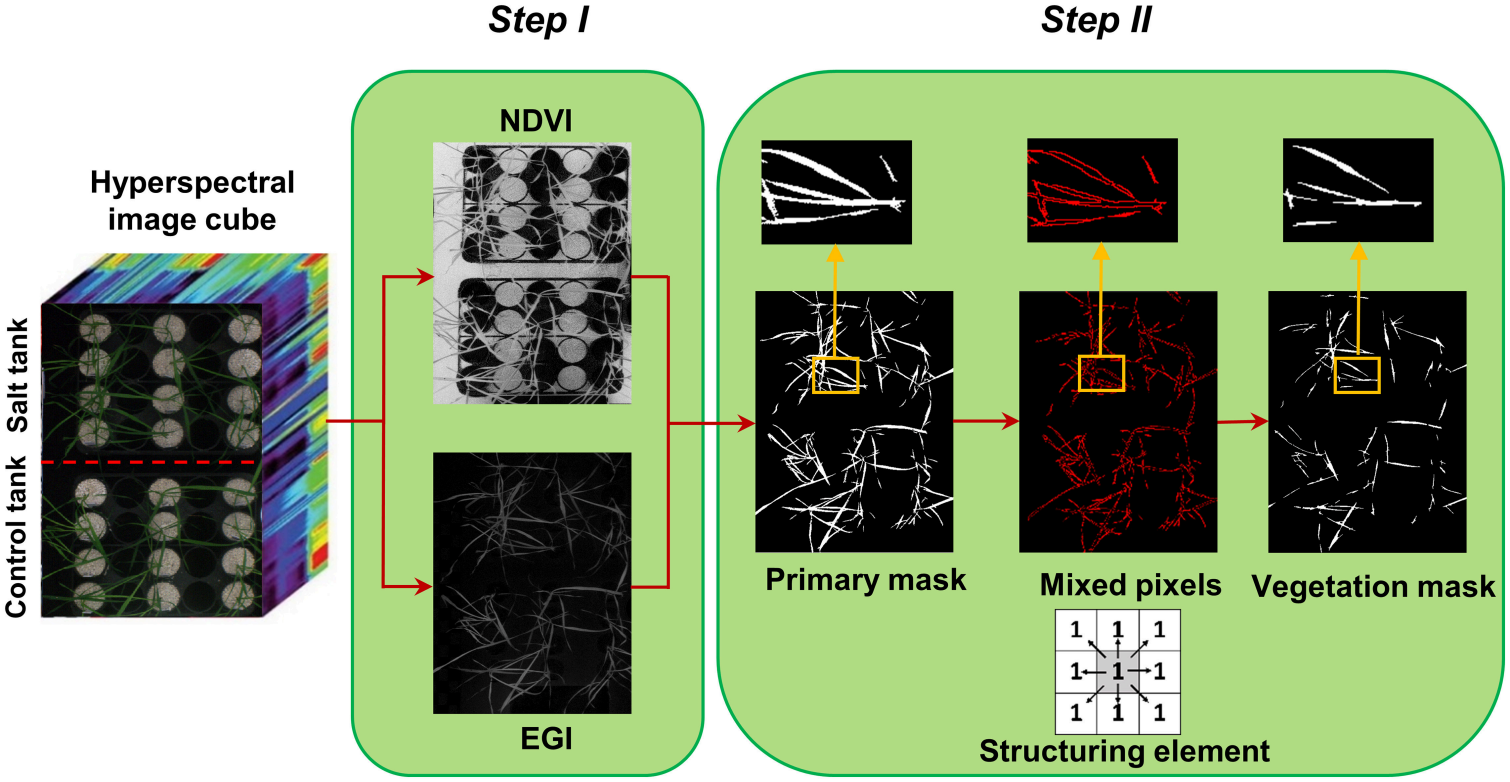
Two steps of creating vegetation binary mask, step I: segmentation of vegetation pixels from background using spectral indices (normalized difference vegetation index and excessive green index), and step II: filtering of mixed pixels at leaf edges using morphological operation (erosion with a 3 × 3 matrix of ones as structuring element).

All tanks were transferred from the Conviron to greenhouse to take hyperspectral images under natural light conditions. To ensure that each hyperspectral image contained both salt and control plants of a single wheat line, individual cone-tainers were removed from the randomized grid and arranged as salt and control tanks, each containing 12 cone-tainers as shown in Figure 1. After imaging, plants were placed back into their original randomized grid positions to avoid confounding effects from changing the tank position during the experiment.

Image acquisition was done ~24 h after salt application when there were no visual symptoms. To reduce the effects of sun angle and shade, images were captured close to noon (i.e., between 11:00 and 13:00 local time). A push broom (along-track scanner) hyperspectral camera (PIKA II, Resonon, Inc., Bozeman, MT 59715, USA) was used for image acquisition, which required constant movement during image capture for two-dimensional spatial information to be accurate. A glide gear slider was used to mount the camera on a horizontal bar. A Dayton DC gearmotor (model: 2L008, Dayton electric Mfg Co. Lake Forest, IL 60045, USA) was utilized to move the slider along at a set speed, with the camera oriented to face downwards. All of this was done as per Moghimi et al. (2017). The camera scanned over 240 spectral channels ranging from 400 to 900 nm with a spectral resolution of about 2.1 nm and captured 640 pixels in the cross-track direction (i.e., perpendicular to the direction of camera motion). The number of pixels in the along track direction was set to 2,000 to assure both control and salt tanks of each line were captured in a single image. Therefore, the pixel size of each hyperspectral image, also known as hyperspectral data cube, was 2,000 × 640 × 240, meaning each pixel has a 240 dimensional feature vector.

The frame rate of the camera was adjusted based on the field of view, the distance between lens and target, and the speed of the camera motion as described by Moghimi et al. (2017). The field of view of the camera lens was 33 degrees, and the distance between the target and lens was about one meter. The speed of the camera was set to 0.025 m/s, thus the calculated frame rate was 27 frames per second to obtain square pixels (aspect ratio of 1:1). Gain and exposure time were adjusted appropriately based on light conditions to avoid over-exposure while taking advantage of the full dynamic range (12 bits).

### Image preprocessing

#### Radiometric calibration

Raw images were radiometrically calibrated to account for non-uniform spatial and spectral responses of the sensor due to variability in gain and offset of each detector. Raw digital numbers (DNs) were converted to radiance (*Wm*^−2^*sr*^−1^*nm*^−1^) using the radiometric calibration file provided by the camera manufacturer. Radiance was then converted to reflectance to normalize image data based on incoming solar irradiance so objects could be compared more objectively across images and across capture dates. A Spectralon panel (Labsphere, Inc., North Sutton, NH, USA) was placed in each image and was used as a reference to convert from radiance to reflectance. Spectralon reflects ~99.7% of incident light equally in all directions regardless of the illuminated light angle. Radiometric conversions were performed using Spectronon Pro software (Resonon, Inc., Bozeman, MT, USA).

#### Noisy band removal

Due to high noise, the first and last five bands were removed prior to any analysis. In addition, spectral bands from 753 to 766 nm and also from 813 to 827 nm were disregarded since they were noisy bands near the O_2_ (~760 nm) and H_2_O (820 nm) absorption regions. Following band removal, 215 of 240 bands were used for analysis. Subsequent analyses were performed using MATLAB R2017a (MathWorks, Inc., Natick, MA, USA).

### Vegetation mask

Segmentation of the target of interest from background is a key step in image analysis. To segment vegetation pixels from background pixels, a binary mask was created by thresholding the normalized difference vegetation index (NDVI) (Rouse et al., 1973) and excessive green index (EGI) (Moghimi et al., 2015). The masks were then multiplied together element-wise to generate a primary mask for leaf segmentation (Figure 1 – step I). Pixels near leaf edges were likely to have spectral characteristics of mixed pixels, because they were located near the vegetation/background boundary. To assure these mixed pixels would not pass the vegetation mask, a morphological operation (erosion with 3 × 3 matrix of ones as structuring element) was applied on the primary mask to check the connectivity of each pixel with its neighbors. Pixels from the primary binary mask that were connected with less than eight neighbors were excluded from the final mask (Figure 1 – step II). This final mask was then used to extract all vegetation pixels from the hyperspectral data cube. The masked hyperspectral data cube was converted to a 2D matrix *X* whose rows were features (i.e., wavelengths) and columns were samples (i.e., pixels) and subsequent analysis was performed on matrix *X*.

### Data analysis

#### Normalized reflectance difference

Matrix *X* was split into two matrices: matrix *C* (*d* × *n*_*c*_) contained only control pixels and matrix *S* (*d* × *n*_*s*_) contained only salt pixels where *d* denotes the number of bands, *n*_*c*_ and *n*_*s*_ represent the number of control and salt pixels from a single hyperspectral image, respectively. The average reflectance for pixels of both treatments (control and salt) was calculated over all wavelengths for each wheat line to determine how the reflectance patterns varied due to salt stress. For this purpose, we proposed a method referred to as the normalized reflectance difference (NRD). For each wheat line, NRD was calculated as follows:

(1) $$\begin{array}{r} NRD_{i}=\;\frac{\left[\frac{1}{n_{c}}\;\sum_{k=1}^{n_{c}}C_{ik}\;\right]-\left[\;\frac{1}{n_{s}}\;\sum_{k=1}^{n_{s}}S_{ik}\;\right]\;}{\left[\frac{1}{n_{c}}\;\sum_{k=1}^{n_{c}}C_{ik}\;\right]\;},\;\text{i}=1,\ldots ,d \end{array}$$

*NRD*_*i*_ represents the difference between the average reflectance of pixels representing control and salt treatments divided by the average reflectance of the control at band *i*.

#### Endmembers extraction

It should be noted that those vegetation pixels that could pass the segmentation steps might not be pure pixels because of limiting factors such as leaf angle, leaf curvature, and shadow. Therefore, to extract the spectral signatures for salt stressed and control plants, the most pure pixels for each class (salt and control class) should first be identified among the pixels passed from the segmentation steps. These pure pixels can be considered as endmembers of the two classes. Each hyperspectral image contained only a single wheat line, but included both salt and control treatments. Consequently, there were only two potential classes and subsequently two respective endmembers in each image. These endmembers are the most spectrally pure pixels in the hyperspectral image. The assumption of pure pixels existence can be correct because of the high spatial resolution we attained (~1 mm).

Based on the strategy proposed by Winter (1999), endmember pixels in a feature space are the vertices of a simplex that has the maximum volume compared to any other simplex formed by other pixels. To elaborate, consider each pixel as a point in a *d*-dimensional feature space where *d* is the number of bands. From prior assumption, there could be *n* endmembers which are pure pixels in the image. These *n* endmembers are the vertices of a (*n*−*1*)-simplex that has the maximum volume in a *d*-dimensional spectral feature space spanned by all pixels (i.e., this simplex contains the majority of pixels in the feature space). Several algorithms and techniques for extracting endmembers based on this idea have been developed with the intention of improving computational time and accuracy (Winter, 1999; Thurau et al., 2010; Chan et al., 2011). To find the unique set of two endmembers comprising the vertices of a 1-simplex in this study, successive volume maximization (SVMAX) was utilized. SVMAX has a modified objective function in which endmembers are identified recursively through a successive optimization problem (Chan et al., 2011). In each image, SVMAX identified two pixels that were the furthest from each other in the high-dimensional feature space, each representing one class: salt and control.

#### Measurement of pixels similarity to endmembers

##### Similarity measurement by solving a quadratic optimization problem

Once endmembers were identified, other pixels were represented as a convex combination of the endmembers. This is a factorization problem in which the matrix of data *X* is factorized as the product of two other matrices, *W* and *H* (Thurau et al., 2010). Matrix *W* contains the endmembers extracted using the SVMAX algorithm, and matrix *H* is the matrix of coefficients. To find *H*, the Frobenius norm of ||*X*−*WH*||_*F*_ can be minimized with two constraints:

(2) $$\begin{array}{r} min\;||X-WH|{|}_{F}\\ s.t.\;\left\{ \begin{array}{l} 1^{T}.\;h_{j}=1\\ \;0\leq h_{ij}\leq 1 \end{array}\right. \end{array}$$

The *j*th column of *X* (*x*_*j*_ : 215 × 1) can be represented as matrix multiplication of *W* (215 × 2) and the vector of coefficients located at the *j*th column of *H* (*h*_*j*_ : 2 × 1), which can be interpreted as the abundance of corresponding endmembers in a particular pixel. These coefficients are most commonly used in spectral unmixing techniques to identify the abundance of each endmember. However, these endmember coefficients are interpreted as the “*similarity*” of a given pixel to the salt and control endmembers in the current study. Therefore, all coefficients should be non-negative and less than or equal to one. Furthermore, the summation of coefficients (each column of *H*) for each pixel needs to be equal to one. Larger coefficients represent more spectral similarity between a given pixel and its corresponding endmember.

The matrix of coefficient *H* can be calculated by solving Equation (3) as a quadratic optimization problem as follows.

(3) $$\begin{array}{r} \underset{h_{j}}{\min}\frac{1}{2}\;h_{j}^{T}Qh_{j}+\;c^{T}h_{j}\;,\;j=1,\ldots ,\;N\\ s.t.\;\{\begin{array}{c} 1^{T}.\;h_{j}=1\\ \;0\leq h_{ij}\leq 1 \end{array} \end{array}$$

where

(4) $$\begin{array}{r} Q=2W^{T}W\\ c=\;-2W^{T}x_{j} \end{array}$$

The algorithm (interior-point-convex) used to solve this optimization problem ran iteratively *N* times, where *N* was the number of pixels. Such an algorithm is not scalable for high-throughput phenotyping on a large scale due to the fact that there are hundreds of images, each containing thousands of pixels, and the algorithm should iteratively run for every single pixel.

##### Vector-wise similarity measurement

**Figure 2:**
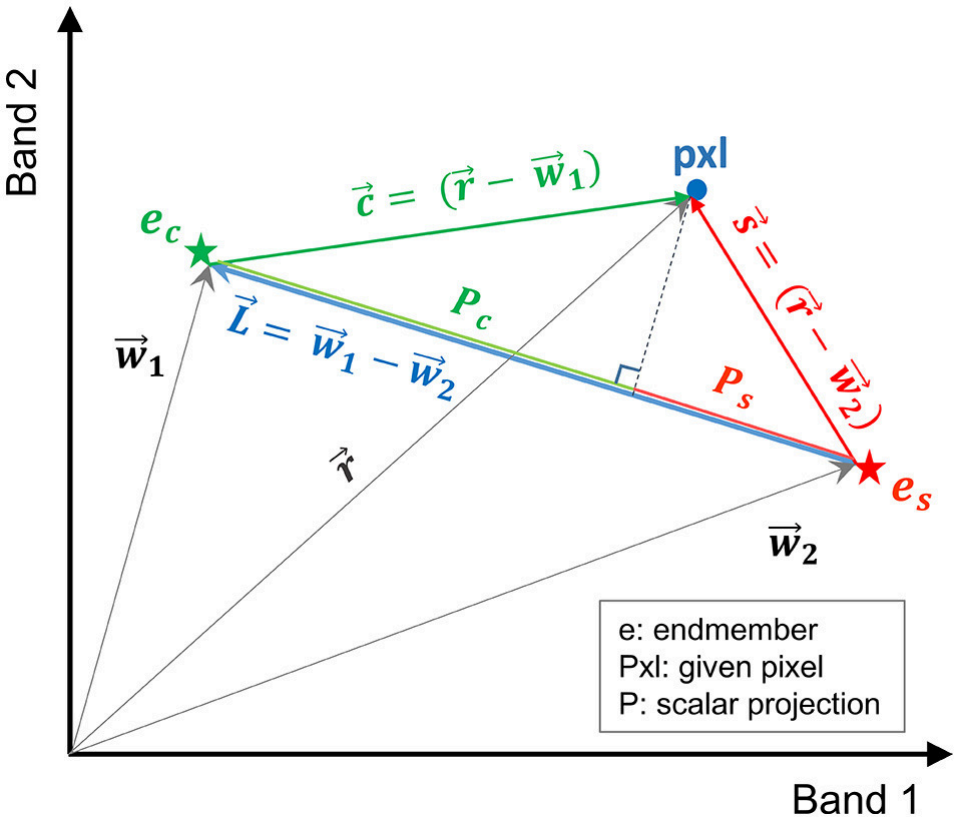
Graphical illustration of the vector-wise similarity measurement (VSM) method to determine the similarity of a given pixel to control and salt endmembers in a 2D feature space.

In this study, a more efficient technique, referred to here as vector-wise similarity measurement (VSM), is proposed to obtain the matrix of coefficients based on the concept that the two endmembers representing the salt and control classes are the two most separated pixels in the feature space. Thus, other pixels can be projected to a line passing between these two endmembers. The distance between the projected point and each of the endmembers can be a measure of similarity, indicating how similar the spectrum of a given pixel is to salt and control endmembers. Figure 2 depicts the graphical illustration of the VSM method. For the purpose of visualization, Figure 2 illustrates the similarity of a given pixel to either of the endmembers only in a two-dimensional feature space spanned by two bands. Note that this technique was implemented in the full dimensional feature space of hyperspectral images.

In Figure 2, ${\vec{w}}_{1}$ and ${\vec{w}}_{2}$ are the vectors of control and salt endmembers, respectively, and $\vec{r}$ is a vector representing a given pixel. The distance between a given pixel and the two endmembers is calculated as follows:

(5) $$\begin{array}{r} P_{c}=\;\frac{\left|\langle c,L\rangle \right|}{\left||L|\right|}\\ P_{s}=\;\frac{\left|\langle s,L\rangle \right|}{\left||L|\right|} \end{array}$$

where $\vec{c}=\;\left(\vec{r}-\;{\vec{w}}_{1}\right)$, $\vec{s}=\;\left(\vec{r}-\;{\vec{w}}_{2}\right)$, $\vec{L}=\;\left({\vec{w}}_{1}-\;{\vec{w}}_{2}\right)$, *P*_*c*_ and *P*_*s*_ are the absolute values of the scalar projection of $\vec{c}$ and $\vec{s}\;$on $\vec{L}$, respectively. In order to impose the constraints similar to Equation (2), *P*_*c*_ and *P*_*s*_ were normalized based on the distance between the two endmembers ($\vec{L}$).

(6) $$\begin{array}{r} D_{c}=\;\frac{P_{c}}{\left||L|\right|}=\;\frac{\left|\langle c,L\rangle \right|}{||L|{|}^{2}}\\ D_{s}=\;\frac{P_{s}}{\left||L|\right|}=\;\frac{\left|\langle s,L\rangle \right|}{||L|{|}^{2}} \end{array}$$

where *D*_*c*_ and *D*_*S*_ are the normalized distance between the projected point of a given pixel to the control and salt endmember, respectively; their summation is equal to one (*D*_*c*_+*D*_*s*_ = 1). Note that similarity *S* can be defined as *S* = *1 – D* (i.e., similarity decreases as distance increases).

The processing time for VSM (~70 s) was ~3,500 times faster than the quadratic optimization algorithm (~0.02 s) using the same dataset (~15,000 pixels) and processing power. It was substantially faster because the similarity of all pixels can be calculated through matrix multiplication rather than going through an iterative loop. In addition, no constraint was needed for VSM, whereas two constraints had to be met in each optimization loop of the quadratic optimization approach.

**Figure 3:**
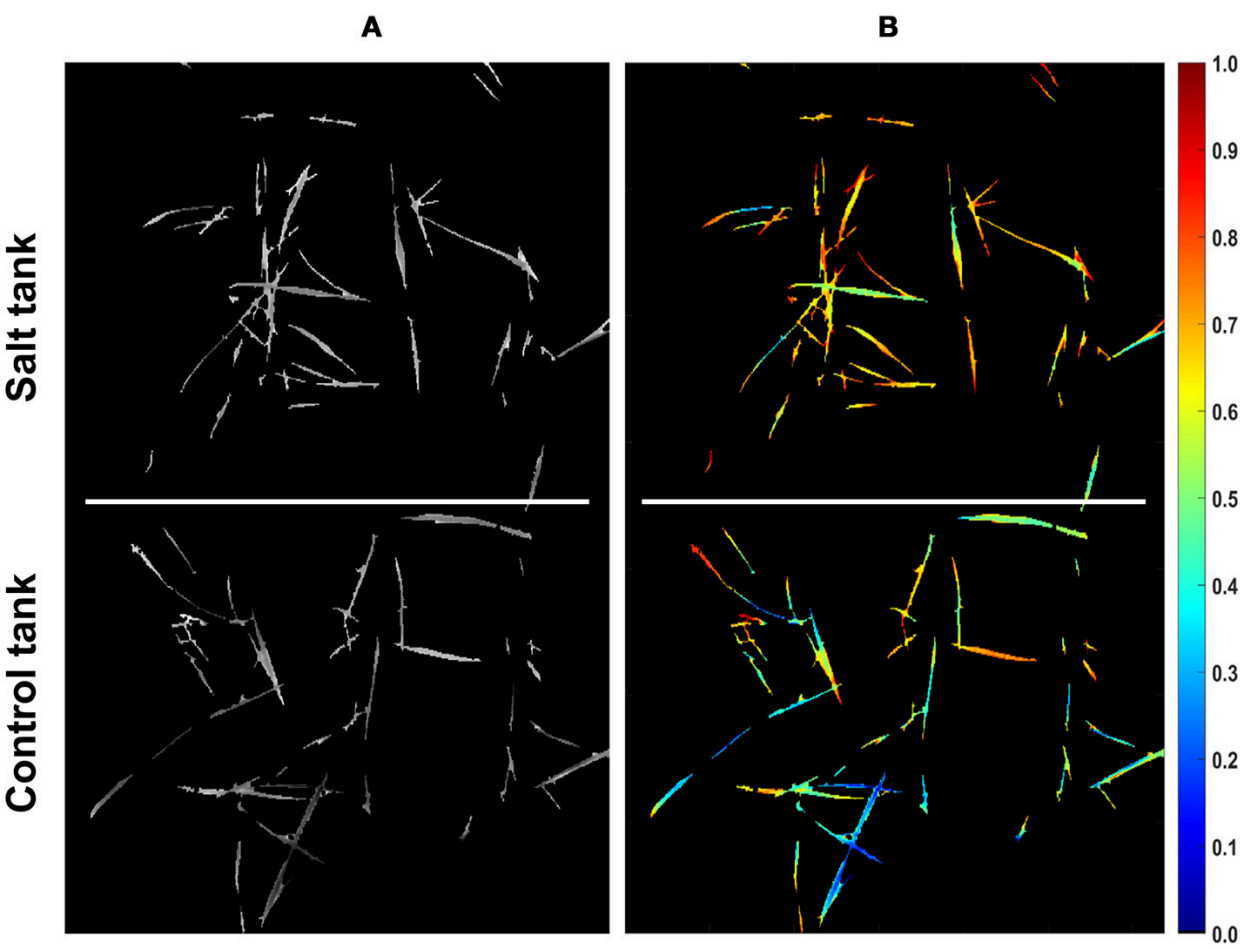
Similarity of pixels to the salt endmember for CS line. **(A)** Similarity of pixels to salt endmember represented as a gray scale image (bright colors denote more similarity). **(B)** Similarity of pixels to salt endmember represented as a colormap (larger values in colorbar denote more similarity).

Since the two distances calculated for pixels complement each other (i.e., they sum up to one), each pixel can be represented with a single value that represents the similarity of a pixel to either of the treatments. Representing pixels in this manner allows dimensionality to be reduced from 215-D to 1-D and more notably, allows for quantification of pixel similarity to either endmember. In this study, the similarity of individual pixels to the salt endmember was extracted as a row vector from the coefficient matrix obtained by VSM for further analyses. The equivalent row and column subscript values corresponding to a single index of similarity vector were determined to assign similarity values to the corresponding non-zero pixels that could pass the mask in a given image. Figure 3 shows the result of mapping from similarity vector of the CS line to a gray scale image. In this image non-zero pixels represent the similarity of control and salt pixels to the CS salt endmember and pixels equal to zero represent the masked background. To illustrate the structure of similarity values in a proper manner, the gray scale image was transferred to a colormap image (Figure 3B).

#### Analysis of similarity vector obtained by VSM

Two methods were used to analyze the similarity vector/gray-scale image obtained by VSM for each line.

##### Histogram distance

The similarity vector obtained by VSM can be represented as a univariate histogram per each class to investigate the distribution of two classes for each line. To assess the tolerance of a wheat line to salt stress, histogram distance between the control and salt class was measured. The histogram distance indicates how much the imposed salt treatment caused a shift in the similarity distribution between the control and salt classes.

A metric distance measure called the minimum difference of pair assignments (MDPA) was used to measure the histograms distance (Cha and Srihari, 2002). MDPA was selected because the type of similarity histogram was ordinal in which order of bins matters. Moreover, similarity/difference between non-overlapping parts of control and salt histograms should be considered in measuring histograms distance. According to Cha and Srihari (2002), MDPA accounts for the similarities of the entire histograms, including overlapping and non-overlapping bins, while other methods such as Bayes error only consider the intersection of histograms for distance calculation. In addition, commonly used methods such as Euclidean distance and Bhattacharyya distance will remain unchanged if we permute the histogram bins. This “*shuffling invariant*” property is not suitable for calculating the similarity of two ordinal type histograms.

MDPA was calculated based on the equation proposed by Cha and Srihari (2002) as follows:

(7) $$\begin{array}{r} D\left(H\left(C\right),H\left(S\right)\right)=\;\overset{b-1}{\underset{i=0}{\sum }}|\overset{i}{\underset{j=0}{\sum }}\left(H_{j}\left(C\right)-\;H_{j}\left(S\right)\right)| \end{array}$$

where *H*(*C*) and *H*(*S*) are the similarity histogram of control and salt classes to salt endmember, respectively; and *b* is the number of bins. In this study, the number of bins was set to 100 and therefore, the width of each bin was 0.01 because the similarity ranged from zero to one.

According to the MDPA equation, the distance between control and salt histograms is the minimum required sample replacements among bins such that the salt histogram becomes identical to the control histogram. This requires that the number of samples (i.e., pixels) be identical for both control and salt class. However, it is rare that the number of samples/pixels belonging to the control and salt classes be equal after initial image processing. To account for the problems associated with an imbalanced dataset, the larger dataset was sub-sampled using a stratified random sampling method in which each bin was randomly sub-sampled based on the ratio between samples of the smaller and larger datasets. The stratified random sampling assured that the distribution of the sub-sampled dataset remained unchanged, which was required for MDPA to achieve proper results. In addition, MDPA, obtained from Equation (7) for each wheat line, was divided by the number of pixels belonging to one of its histograms (either salt or control histogram since they have equal number of pixels after subsampling) to account for the difference between the numbers of pixels among the wheat lines.

##### Bayesian inference

After sub-sampling, the gray-scale image obtained by VSM per each wheat line had equal number of salt and control pixels, each representing the similarity to the salt endmember. To make inferences about the tolerance of each wheat line using these gray-scale images, the posterior probability of salt class was calculated using Bayesian inference. The framework of the Bayesian inference is Bayes' rule, which was written in this study as:

(8) $$\begin{array}{r} P\left(salt|x\right)=\frac{P\left(salt\right)\times P\left(x|salt\right)}{P\left(x\right)}\;\\ =\frac{P\left(salt\right)\times P\left(x|salt\right)}{P\left(salt\right)\times P\left(x|salt\right)+P\left(control\right)\times P\left(x|control\right)} \end{array}$$

where *P*(*salt*|*x*) is the posterior probability of salt class given an observation *x*. *P*(*x*|*salt*) is the class-conditional probability which is the conditional probability of observation *x* given salt class, and *P*(*x*) is the evidence representing the occurrence probability of observation *x*. *P*(*salt*) is the prior probability, and it was calculated as the ratio of the number of salt pixels to the total number of vegetation pixels in a single image. The posterior would have been biased toward the larger class if the dataset were imbalanced, and this is why stratified random sampling was performed in an earlier step. After sub-sampling, each class had an equal number of pixels, and therefore, an equal prior [*P*(*salt*) = *P*(*control*) = 0.5]. As a result, Equation (8) can be simplified by canceling out the equal prior of salt and control classes from the numerator and denominator.

In this study, the similarity of pixels to the salt endmember, denoted as *s*, was considered as the observation *x*. To compute the class-conditional probability for salt and control classes, the similarity values of each class, which were continuous, were discretized into two ordinal bins by specifying 0.5 as the split point. Pixels with more similarity to the salt endmember (*s*>0.5) were categorized in one bin and pixels with more similarity to the control endmember (*s* ≤ 0.5) were categorized in the other bin. Afterward, the posterior probability of salt class for pixels with more similarity to the salt endmember [i.e., *P*(*salt*|*s*>0.5)] was calculated for each of the wheat lines as follows:

(9) $$\begin{array}{r} P\left(salt|s>0.5\right)=\;\frac{P\left(s>0.5|salt\right)}{P\left(s>0.5|salt\right)+\;P\left(s>0.5|control\right)} \end{array}$$

Equation (9) was derived from Equation (8) by canceling out the equal prior of salt and control classes and considering *s* > 0.5 as the observation. The numerator of Equation (9) is the fraction of salt pixels with more similarity to the salt endmember to the total number salt pixels, and the denominator represents the fraction of all vegetation pixels with *s* > 0.5 to the total number of vegetation pixels within a single image.

For a susceptible line, the spectral response of pixels representing the salt stressed plants become more district from that of the control plants because of physiological and metabolic alteration in stressed plants. As a result, the salt pixels shift more toward the salt endmember and become more distinct from the control endmember; and hence, the number of similar pixels to the salt endmember among salt treatment pixels [*P*(*s* > 0.5|*salt*)] is much larger than the number of pixels similar to the salt endmember among control pixels [*P*(*s* > 0.5|*control*)]. Therefore, it can be inferred that a susceptible line should have a larger posterior probability of salt class [*P*(*salt*|*s* > 0.5)] than a tolerant line in which a given pixel with more similarity to the salt endmember can be assigned to the salt class with a lower confidence as its posterior probability of salt class is relatively lower.

## Results

### Conventional biomass measurement to assess salt tolerance

The primary objective of this research was to quantitatively rank wheat lines based on salt tolerance using HSI. As a case study, four *Triticum aestivum* bread wheat lines (see Methods for full description) were selected for assessment of salt tolerance with destructive biomass measurements in parallel with HSI.

Many different molecular, physiological, and growth parameters can be used to assay salt tolerance differences between genotypes, including Na+ uptake, the ratio of K/Na+, photosynthetic activity (Nxele et al., 2017), gene expression (Agarwal et al., 2013), and aerial and root biomass in salt versus control conditions over extended growing periods (Munns and James, 2003). To assess salt tolerance, salt treatments were applied for 2 weeks, and then root and aerial biomass were measured on a dry weight basis (see Methods).

According to previous studies, Kharchia is a salt tolerant line since it maintains a stable harvest index and yields well in high salt conditions (Schachtman et al., 1992; Munns et al., 2006), while CS is a salt-sensitive cultivar (Zhang et al., 2016). Therefore, the main objective of performing conventional phenotyping was to identify the tolerance of the two unknown additional alloplasmic lines, co(CS) and sp(CS). The results of biomass measurements for these two lines were compared with CS since they contain the exact same nuclear background as CS, which allowed for a direct comparison of biomass to CS.

**Figure 4:**
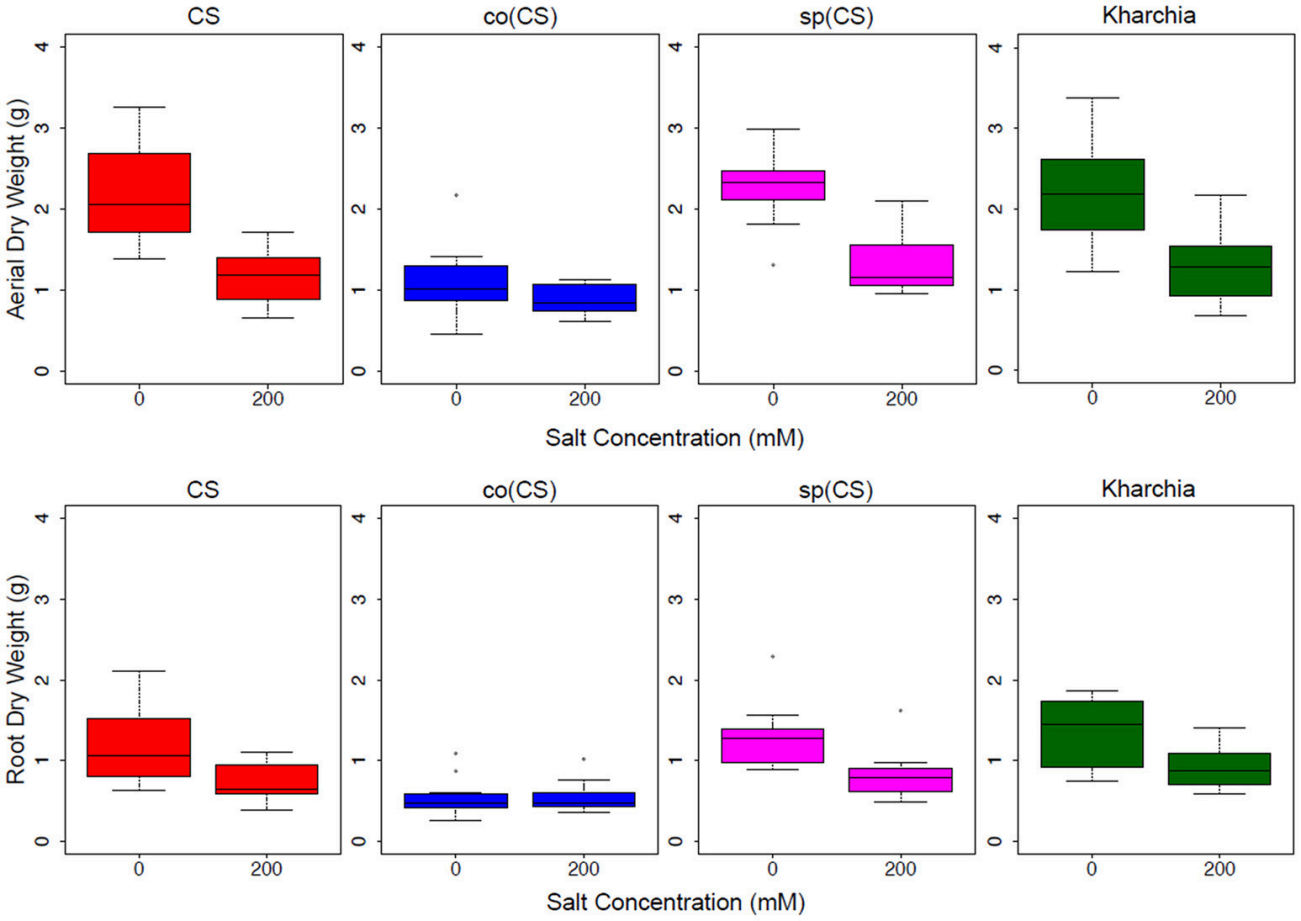
Aerial and root biomass in control (left bars) and salt (right bars) conditions. The upper panel shows aerial biomass and the lower panel shows root biomass for Chinese Spring (CS), the two alloplasmic lines *Ae. columnaris*(CS) [co(CS)] and *Ae. speltoides*(CS) [sp(CS)], and Kharchia.

The biomass measurements revealed that both CS and sp(CS), unlike co(CS), showed a reduction in both aerial and root biomass after growth in the presence of 200 mM NaCl (Figure 4). The analysis of variance found significant interactions at all levels for aerial and root biomass, including between salt level and genotype (Supplemental Table 1). A closer examination of effect sizes using linear mixed modeling showed significantly less change in aerial and root biomass from 0 to 200 mM in co(CS) when compared to CS (Supplemental Table 2), indicating that the alloplasmic line co(CS) is more salt tolerant than the nuclear donor CS in terms of salt effect on biomass. However, overall growth rate may be impacted in co(CS), as biomass in the absence of salt is less than that of CS. A possible explanation for this observation is that altered nuclear-cytoplasmic communication in this line could lead to improper expression of organellar (or nuclear) transcripts involved in stress tolerance, therefore “priming” the alloplasmic for stress and reducing sensitivity to salt stress (as measured by the difference in biomass between 0 and 200 mM).

The response of the other alloplasmic line [i.e., sp(CS)] was not significantly different when compared to CS (Supplemental Table 2); however, it trended toward less change in response to salt compared to CS for aerial biomass. The change in root biomass was almost identical to that of CS. Based on these observations, it can be inferred that sp(CS) is slightly more tolerant than CS.

Although it is historically known that Kharchia is more tolerant than CS, the result of biomass measurements of Kharchia was also compared with CS to examine if the conventional biomass measurement could capture the difference between these two lines with dissimilar genome backgrounds. Intriguingly, the magnitude of aerial biomass change between the control and treatment in the highly salt-tolerant Kharchia cultivar was not significantly different when compared with salt-sensitive CS (Figure 4 and Supplemental Table 2). However, similar to sp(CS), the trend was also toward a smaller change in response to salt than CS. This indicates that biomass measurement, although a convenient parameter to measure in a lab environment, may not always reflect the actual field performance in desirable traits such as harvest index or yield. This is consistent with previous results that showed a substantial biomass decrease for Kharchia in the presence of salt, yet also a high relative yield and harvest index (Schachtman et al., 1992). Without the substantial historical knowledge of how Kharchia was derived from Indian landraces adapted to sodic soils (Munns et al., 2006), the assessment of salt tolerance with hydroponic screening and biomass measurement for this study may have missed this highly valuable source of germplasm.

Based on the results of our conventional salt tolerance and historical knowledge, we can conclude that Kharchia and co(CS) are more salt-tolerant than sp(CS) and CS. In addition, the time-consuming and laborious process of conducing the conventional biomass measurement for salt tolerance assessment underscored the need for more informative and quantitatively precise screening techniques to rapidly and non-destructively assess salt tolerance, particularly when comparing cultivars with drastically different genetic backgrounds and growth regimes, such as Kharchia and CS that differ in vernalization requirements and photoperiod sensitivity (Koebner et al., 1996). However, since co(CS) and sp(CS) have identical nuclear backgrounds to CS but only differ in their organellar genomes, direct comparisons of biomass are more valid.

### Normalized reflectance difference

**Figure 5:**
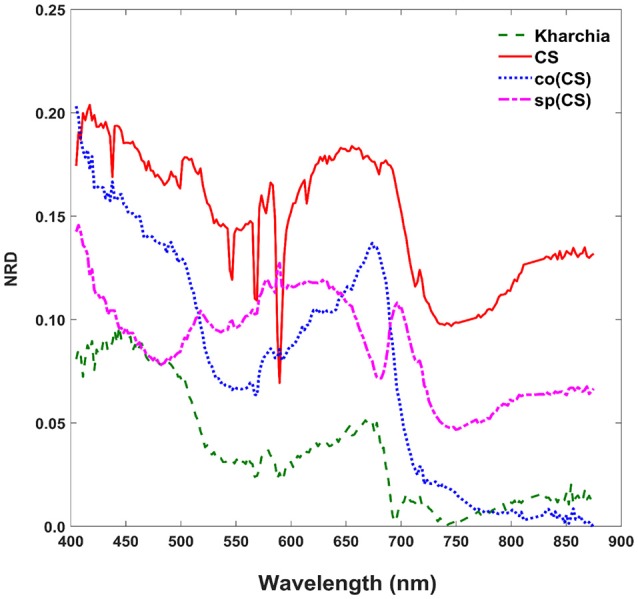
Normalized reflectance difference (NRD) of wheat lines, indicating how much the averaged reflectance of the salt pixels changed compared to the control pixels in response to the imposed salt stress on each wheat line.

**Figure 6:**
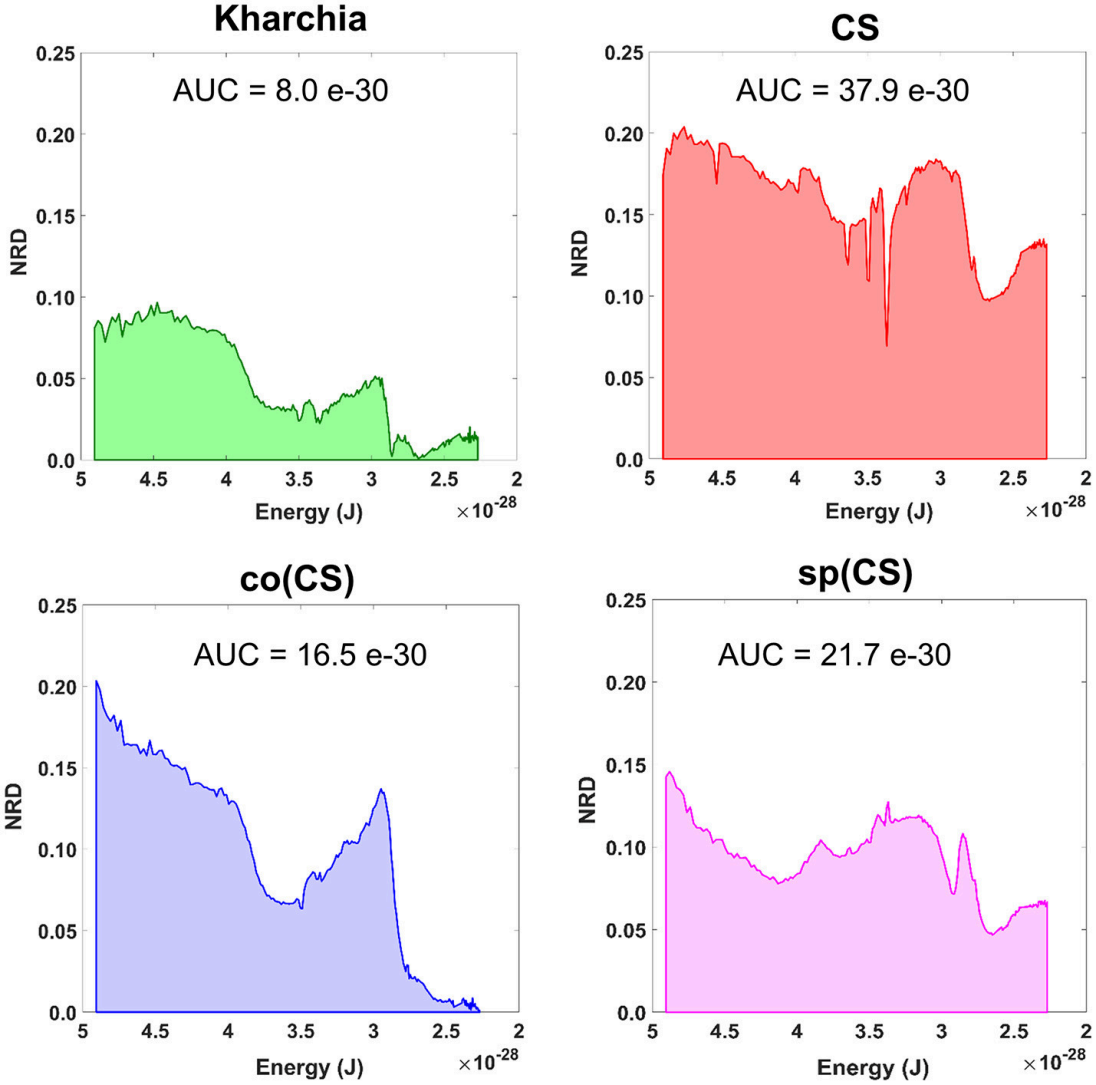
Area under the normalized reflectance difference (NRD) curve calculated as the summation area of all trapezoids formed between two successive bands and the NRD curve [wavelength (nm) on x-axis was converted to energy (J) using the Planck equation].

To gain an overall view of how the four wheat lines differed in response to salt-stress, normalized reflectance difference (NRD) was examined. NRD in Equation (1) indicates how much the reflectance of the salt class changed compared to the control class in response to the imposed stress (Figure 5). According to the NRD, Kharchia is the most tolerant among the four lines, which is consistent with previous studies and historical knowledge (Schachtman et al., 1992; Munns et al., 2006) but inconsistent with assessment of salt tolerance with biomass measurement. On the other hand, CS appears to be the most susceptible due to the larger difference between average reflectance of control and salt pixels. This observation was consistent with both previous studies (Zhang et al., 2016) and biomass measurement in this study. Based on NRD, the two alloplasmic lines, co(CS), and sp(CS), are somewhere in between Kharchia and CS. The NRD method alone provides a rapid and brief insight to identify the tolerant lines, but not in a quantitative manner. A quantitative ranking can be generated by calculating the area under the NRD curve as the summation area of all trapezoids formed between two successive bands and the NRD curve. To give more physical meaning to the area under NRD, wavelength (nm) of the x-axis was converted to energy (J) using the Planck equation. Consequently, area under the curve (AUC) can be presented in the unit of energy. The results of AUC for the wheat lines are shown in Figure 6. The order of lines in terms of their tolerance to salt based on AUC is similar to the results achieved by conventional biomass assessment, except that we could detect the strong salt tolerance of Kharchia more readily with AUC. It is also notable that both alloplasmic lines show smaller AUC than the CS nuclear donor.

### Endmembers extraction using SVMAX

**Figure 7:**
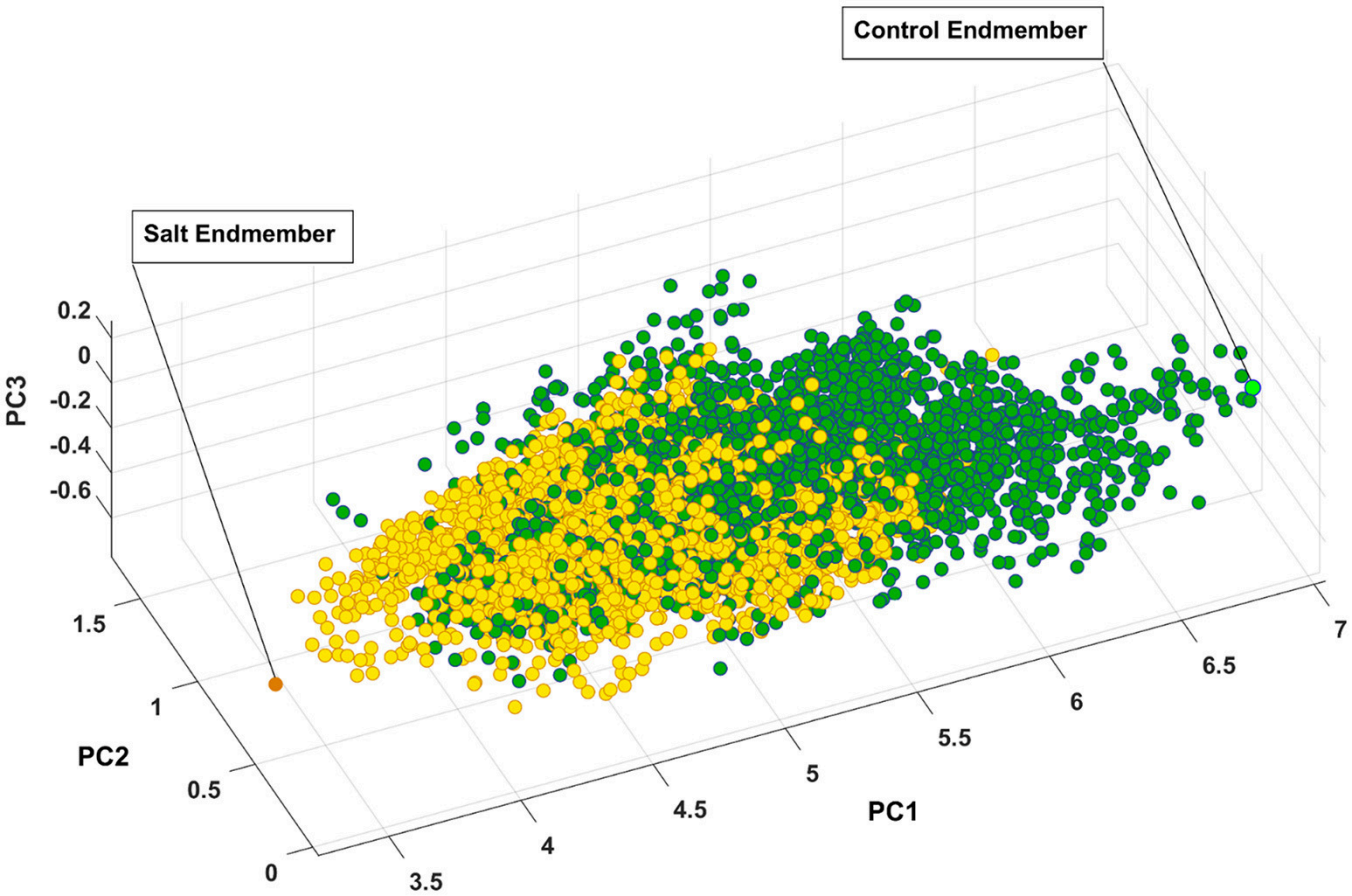
Control and salt endmembers identified by SVMAX for CS line as the furthest away pixels form each other illustrated in a space spanned by the first three principal components (i.e., PC1, PC2, and PC3). Green and yellow circles represent control and salt pixels, respectively.

Figure 7 presents the salt and control endmember locations of CS with respect to other pixels where all of the pixels extracted to find endmembers have been projected onto the first three components of principal components analysis for visualization purposes. It is evident that the endmembers identified by SVMAX are the pixels located at the extremes in the space spanned by the first three principal components.

For this study, the endmembers are assumed to be the pixels that represent either the control or salt treatments. Afterwards, the reflectance of other pixels can be represented as the similarity to either of these endmembers while reducing the dimensionality from 215 to one.

Figure 7 illustrates how pixels belonging to each of the two classes are intertwined (i.e., many occupy similar feature space) while a slight shift toward the lower values of PC1 can be observed. One explanation for this intertwinement is that reflectance of a plant is not only a function of health status but it also depends on other parameters such as leaf angle, geometry of leaf, and configuration of sensor, leaf surface, and light source. As a result, we focused on the distribution of similarity to salt endmember for control and salt pixels rather than a pixel-by-pixel analysis.

### Histogram distance measure

**Figure 8:**
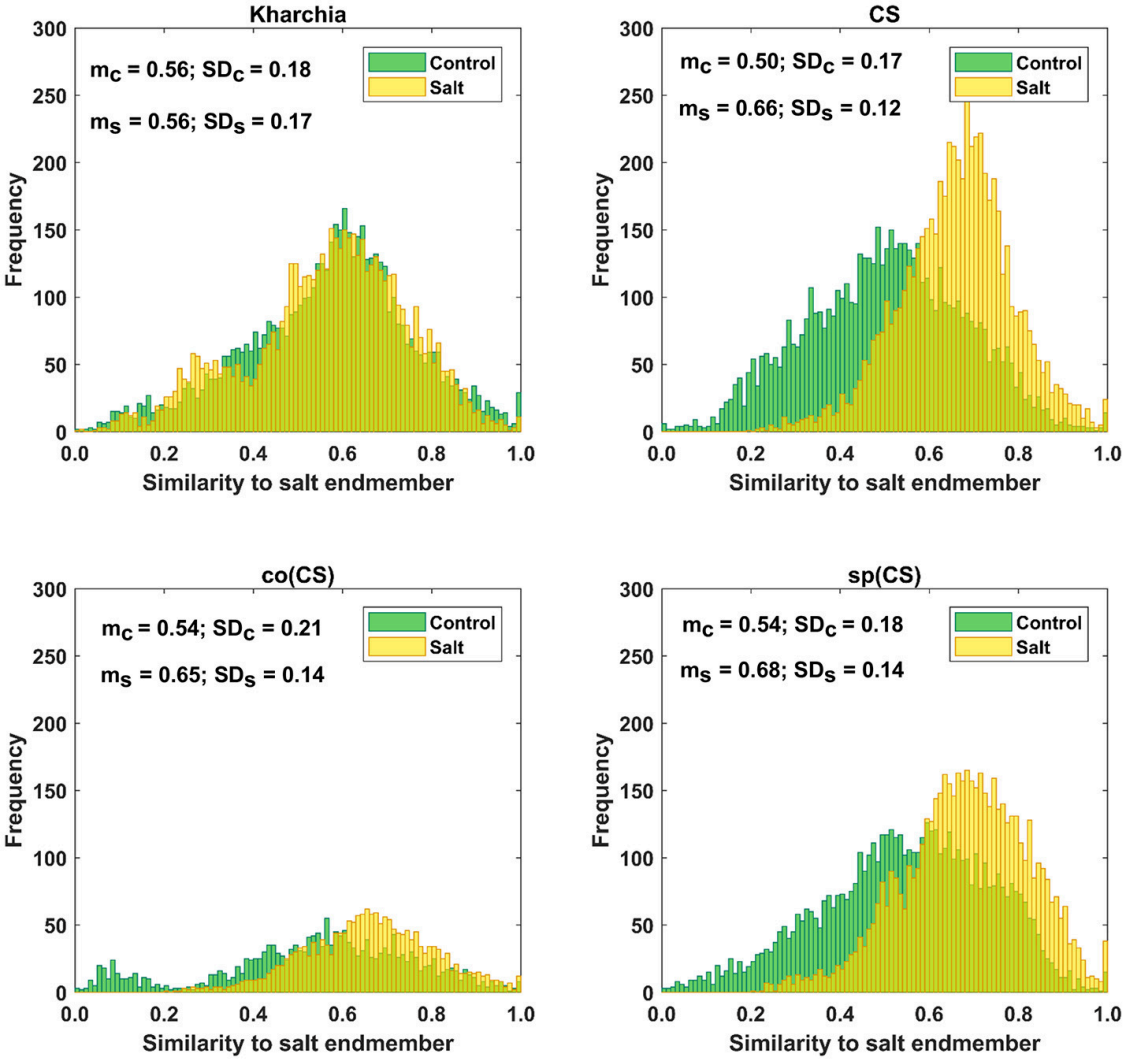
Histogram of similarity to salt endmember for control and salt pixels of the four wheat lines. Kharchia, as a tolerant line, and CS, as a susceptible line, have the smallest and largest mean shift of salt pixels (mc) toward the salt endmember, respectively.

The distributions of pixel similarity to the salt endmember for control or salt treatments are illustrated via histogram (Figure 8). CS had the largest mean shift of salt pixels toward the salt endmember from *m*_*c*_ = 0.50 to *m*_*s*_ = 0.66 indicating that it is the least tolerant line. It is also notable that the standard deviation decreased with salt induction causing the pixels to aggregate toward the salt endmember. In the similarity distribution of the salt stress class of CS, there were no pixels similar to the control endmember.

For Kharchia, the mean of the salt pixels' similarity to the salt endmember remained approximately unchanged compared to the control pixels (*m*_*c*_ ≅ *m*_*s*_), and the standard deviation changed slightly (Figure 8). This suggests that the distribution of control and salt pixels between the two endmembers was similar for both conditions.

The mean of salt pixels' similarity to the salt endmember for co(CS) and sp(CS) shifted to the larger value indicating that salt stress caused the spectral response of pixels to change. However, this shift was lower compared to CS line in which salt stress made a large difference between the control and salt distribution.

Except for Kharchia (the most tolerant wheat line), salt stress caused the similarity distribution of pixels belonging to the salt class to shift toward the salt endmember and caused the standard deviation to decrease.

**Figure 9:**
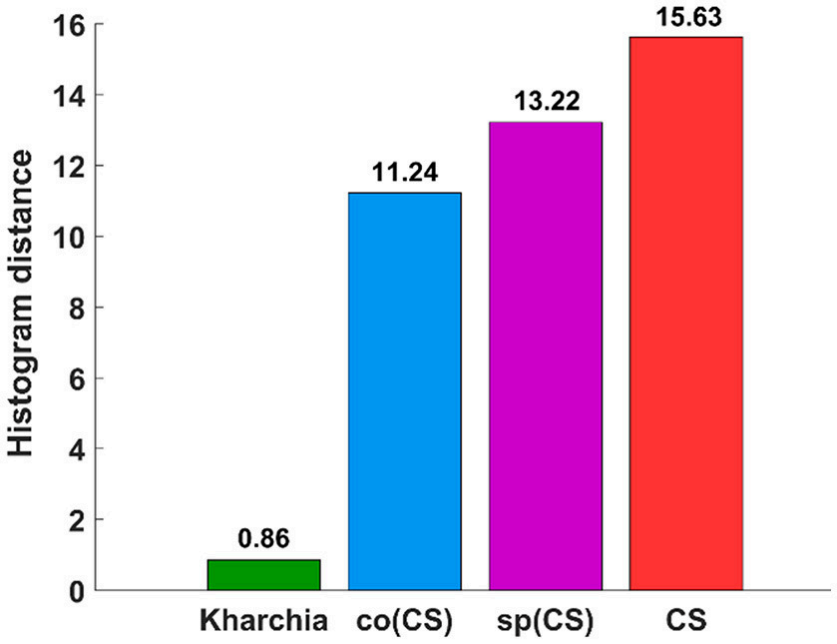
Results of the minimum difference of pair assignments (MDPA) for calculating histogram distance between distribution of similarity to salt endmember for control and salt pixels.

To quantify the histogram shift due to the imposed salt stress, MDPA was calculated based on Equation (7). Based on MDPA, CS has the largest distance between its salt and control classes, whereas Kharchia has the lowest distance (Figure 9). Except for Kharchia, that has been discussed ad nauseam, the results of MDPA for ranking wheat lines are similar to the results of conventional biomass measurement.

### Posterior probability

To provide an additional method to quantify salt tolerance of lines, we also calculated the posterior probability of salt class for each line if a given pixel is more similar to the salt endmember [i.e., *P*(*salt*|*s* > 0.5)]. Table 1 presents the posterior probability, prior, class-conditional probability, and evidence for each wheat line. As expected, CS had the largest posterior probability. This indicated that if a pixel of CS was more similar to the salt endmember, then this pixel could be classified as salt class with a higher confidence in comparison with other lines. The relatively large posterior probability of salt class for CS also implied that the spectral response of salt treated plants became more distinct than control plants even after a short time (only one day after salt application) such that the salt pixel were more similar to the salt endmember and more distinct from the control pixels. This can be attributed to the susceptibility of CS to salt stress. However, no such inference could be made for Kharchia because pixels with more similarity to the salt endmember could not be classified with high confidence to salt class. In addition, the subtle difference of posterior and prior probabilities of Kharchia implied that the imposed salt stress did not have a significant impact on the spectral response, and hence, the salt and control pixels of Kharchia were alike in terms of similarity to the salt endmember.

**Table 1:** Bayes' rule components (prior, class-conditional probability, evidence, and posterior) for wheat lines.

	***P* (salt) (%)**	***P* (*s* > 0.5|salt) (%)**	***P* (*s* > 0.5) (%)**	***P* (salt|s > 0.5) (%)**
Kharchia	50.00	68.61	68.32	50.21
CS	50.00	89.77	70.88	63.33
co(CS)	50.00	86.21	74.84	57.60
sp(CS)	50.00	89.32	75.70	59.00

The quantified ranking of wheat lines based on Bayes' rule is consistent with the MDPA ranking, and similar to the results of biomass measurement. Again, we could readily detect the robust salt tolerance of Kharchia compared to CS, which was missed with conventional phenotyping. We could also quantify differences between the nuclear donor CS and the two alloplasmic lines, co(CS) and sp(CS). The result of posterior probability indicates that HSI could be useful to measure subtle changes which are less noticeable with conventional phenotyping.

**Figure 10:**
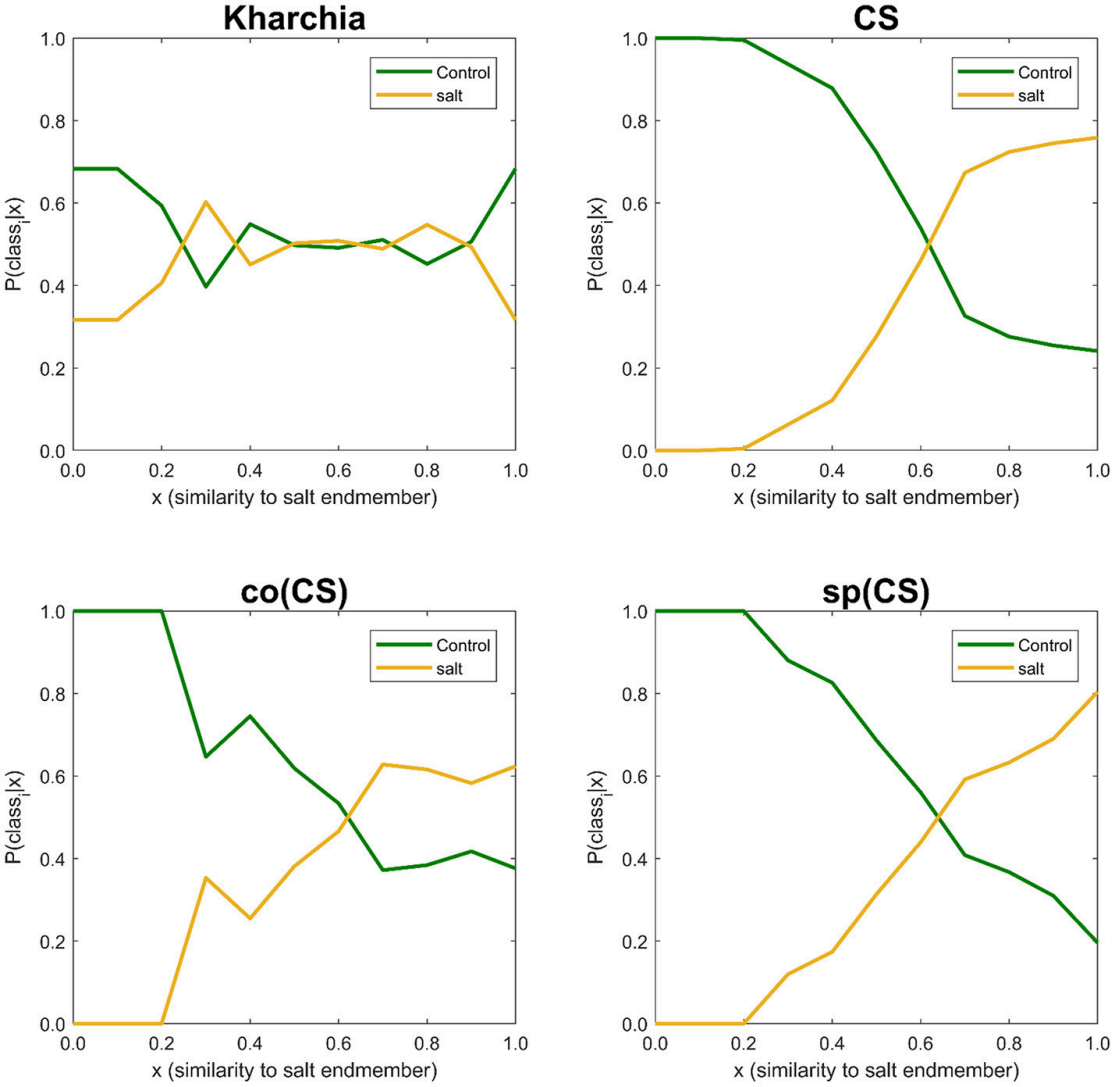
Posterior probability of belonging to control and salt classes given similarity to salt endmember. Posterior probability was calculated for each individual 10 bins of similarity to salt endmember.

The similarity values were also discretized into 10 ordinal bins to observe how the posterior probability of both salt and control class vary over the variation range of similarity to the salt endmember (i.e., *posterior*_*i*_ = {*P*(*class*_*i*_|*s*_*j*_) *s*.*t*. *s*_*j*_ ∈ *bin*_*j*_, *j* = 1, …, 10}, *and i*∈{*control, salt*}) (Figure 10). The distribution of posterior probability of the two classes for Kharchia, the most tolerant line, represented a high degree of uncertainty in making inference because posterior probabilities of the two classes fluctuated throughout the similarity bins. This fluctuation can be associated with the absence of significant difference in spectral response of control and salt pixels due to the high degree of tolerance to salt stress in Kharchia. For the other wheat lines, the posteriors of two classes intersect at one point, which can be considered as the threshold of decision for making inferences. For co(CS), the second most tolerant line, posteriors fluctuated after the threshold point, such that the posterior of salt class is highly likely indicating the moderate tolerance of co(CS) line to salt stress. However, for CS and sp(CS), the two most susceptible lines, posterior probability of control and salt classes diverged after the decision point. In general, *P*(*salt*|*s*_*j*_) of these two susceptible lines monotonically increased as similarity to the salt endmember increased. The trend of posteriors for these two wheat lines indicated how salt pixels became more distinct than control pixels in response to the imposed salt stress.

**Figure 11:**
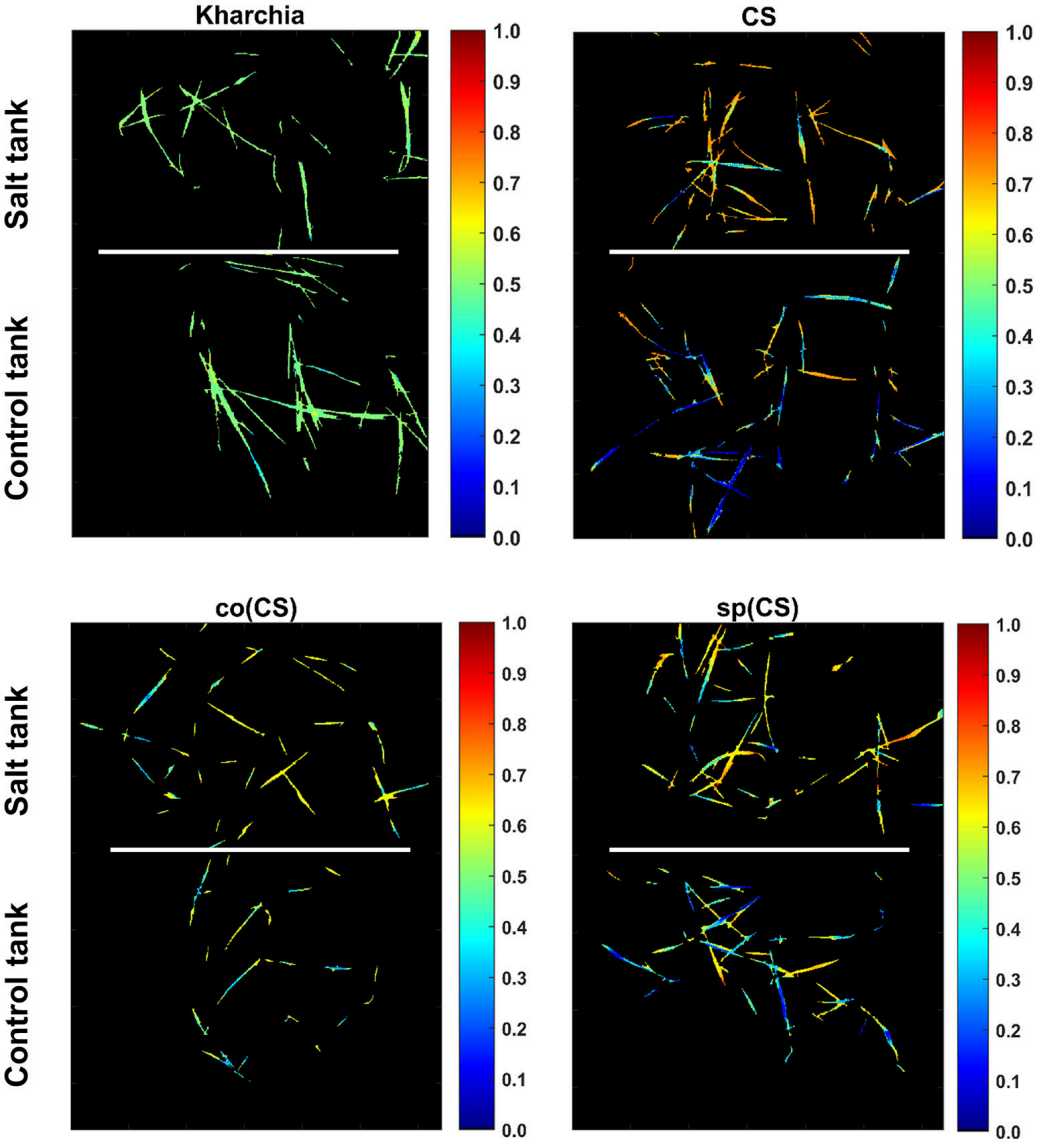
Posterior probability of belonging to salt class given similarity to salt endmember for control and salt pixels of each wheat line [i.e., P(salt|s)].

To illustrate the result of posterior probability more intuitively, *P*(*salt*|*s*_*j*_) for all pixels in each wheat line image is shown as a colormap in which a distinct color was used to highlight the difference between posterior probability of pixels in control and salt regions (Figure 11). According to Figure 11, the control and salt pixels of CS, the most susceptible line, can be visually distinguished, whereas the majority of Kharchia pixels, regardless of belonging to either class, have a similar posterior probability.

## Discussion

Three methods including area under the NRD curve, MDPA, and posterior probability were utilized in this study to analyze hyperspectral images of wheat lines. In all methods, salt stress treatment of each line was compared to its control treatment because differences in spectral responses of a given line may not necessarily be related to the imposed stress, but rather to differences in inherent characteristics such as having waxy and/or darker leaves. The order of ranking of the examined wheat lines was similar for all of these methods. Kharchia was ranked as the most tolerant line followed by co(CS) and sp(CS). CS was identified as the most susceptible line by all three methods. In addition to ranking the wheat lines, more inferences could be made from calculating the posterior probability compared to the other two methods. For instance, it could provide the ability to observe the variations of posterior probability over all similarity bins (Figure 10). This observation can be used to define a threshold of making a decision for classification purposes if the classification of salt and healthy plants is of interest.

In this section, results and achievements of this research study are discussed.

### Quantitative ranking

Our findings revealed that conventional phenotyping methods to identify salt tolerant wheat lines could be replaced by the fast and non-invasive methodology proposed in this study. It was surprising to find that the conventional assessment of salt tolerance with biomass measurement was not consistent with the anecdotal and historical evidence of salt tolerance for the Indian landrace Kharchia. However, biomass measurements of Kharchia were indeed consistent with previous studies that documented a significant biomass decrease, yet stable harvest index and yield in response to salt stress (Schachtman et al., 1992). Kharchia does show a significantly reduced level of Na+ uptake in the 5th leaf when compared to salt-sensitive cultivars, which likely explains the stable harvest index of this line (Schachtman et al., 1992). Based on these contradictory findings, it appears that the most informative way to conventionally assess salt tolerance is to measure multiple growth and physiological parameters simultaneously. However, this is laborious, expensive, and not feasible for many lines or populations simultaneously as is required for breeding programs. In contrast, our method could be used to quantitatively and objectively rank salt tolerance of individual wheat lines in a non-destructive and cost-effective manner. Moreover, the proposed method could be successfully used to detect subtle differences between lines, such as between alloplasmics and euplasmics.

### Early detection

Detection of tolerant wheat lines was achieved as early as one day after the salt treatment when no visual symptoms were observed, and physiological and growth measurements were not yet possible. Early detection enables faster screening cycles and reduces the energy and costs needed to maintain plants in a controlled environment. The findings of this experiment provide evidence that breeders and plant geneticists would be able to properly manage time, energy, cost, and space in greenhouses while maintain accuracy and improve precision by implementing HSI and the proposed analytical methods. Faster assessment of stress tolerance is a major advantage to breeding programs and basic research alike.

### Analysis of complex and high dimensional hyperspectral images

Appropriate preprocessing tasks and machine learning techniques were utilized to leverage the full potential of HSI in a phenotyping context. The achievements of this study in the context of hyperspectral image analysis are discussed in the following sub-sections.

#### Elimination of mixed pixels

A mixed pixel contains spectral information derived from a combination of objects that inherently have varying spectral characteristics. Mixed pixels are often located along the edges of objects in images, especially when the spatial resolution of an image is finer than the individual objects contained in the image (e.g., a leaf). In this study, mixed pixels along the edges of leaves were eliminated from the binary vegetation mask using morphological image processing. Without masking, these mixed pixels would have led to reduced accuracy through the endmember selection and subsequently in computing the similarity of pixels to the salt endmember on which further analyses were based. Prior to using morphological operations, filtering based on vegetation indices allowed us to appropriately mask pixels that were covered by shadows from other leaves or objects.

#### Interaction between incident light and leaf surface

The interaction between incident light and the leaf surface is complex due to leaf angle and curvature. The intensity of the reflected light from leaves is both a function of physiology and leaf geometry, and tends to cause a significant change in the spectral response of vegetation. Behmann et al. (2015) reported 60% of the spectral response of sugar beets could be influenced by the geometry of plant leaves. This provides explanation for observing several control and salt pixels having similar spectral properties (pixels were not perfectly distributed in two well-separated classes). Although we did not explicitly address the issue of leaf geometry, we could mitigate its negative effects on the results by considering the similarity distribution of pixels to the salt endmember. By deploying a fuzzy concept (i.e., where each pixel can be partially similar to either endmember) rather than a hard classification (i.e., binary classification where each pixel is a member of only a single class), downstream processing techniques were possible, such as posterior probability and histogram distance.

#### Complex interpretation of hyperspectral images

In this research, the curse of dimensionality in analysis of hyperspectral images was addressed by projecting from a high dimension (215-D) feature space onto one dimension. The one-dimensional feature space, which can be represented as a gray-scale image, denoted the similarity of each pixel to the salt endmember while maintaining the required information for making further inferences. As a result, interpretation of hyperspectral images could be performed in a much more efficient manner with more meaningful information. By reducing dimensionality, results are simpler and are easier to comprehend. Because agricultural research groups are oftentimes comprised of many scientific disciplines, this directness is imperative. In general, the proposed strategy helps to interpret complex hyperspectral images by discovering the underlying hidden features caused by salt stress. This strategy will also provide more flexibility in selecting ML methods to analyze images. For instance, MDPA was utilized to measure the distance between the similarity distributions of control and salt pixels to the salt endmember. Another benefit of conversion to a one-dimensional feature space is that visualization of relevant spectral information is possible in a more meaningful manner while involving all bands in similarity measurement and maintaining spatial integrity.

The other important advantage is that the required computational time and data storage space for analyzing and storing hyperspectral data was significantly reduced due to converting from a high-dimensional to one-dimensional space. For instance, the average size of a hyperspectral image (2000 × 640 × 215) was ~400 MB while the size of obtained gray scale image (2000 × 640 × 1) was less than 1 MB. It is also noteworthy that the VSM method used for computing the coefficient matrix proposed herein significantly reduced the processing time compared to the quadratic optimization problem that is commonly used.

## Conclusion

In this study, we investigated the application of hyperspectral imaging in assessment of salt stress in four wheat lines one day after salt treatment. A novel pipeline was proposed for hyperspectral image analysis to leverage the full potential of HSI in a salt stress phenotyping context. The proposed pipeline can be applied for other plant phenotyping traits. The results of this study demonstrated the feasibility of quantitative ranking of wheat lines based on their salt tolerance by integrating HSI and novel analytical approaches. Quantitative and objective ranking methods are much needed, and provide invaluable information that can accelerate genomics research, such as GWAS or QTL mapping, used in breeding programs. The quantitative ranking of salt stress tolerance helps breeders integrate salt tolerance results with other desired traits (e.g., grain yield), which ultimately accelerates the development of new plant varieties. In future work, we will focus on identifying sensitive wavelengths to aid in development of a multispectral camera for salt stress phenotyping, and consequently reducing the cost of the sensor as well as the complexity of data collection associated with a hyperspectral line scanner.

## Data availability statement

The datasets generated and analyzed during the current study are available in the Data Repository for University of Minnesota at https://doi.org/10.13020/D69Q3K.

## Author contributions

AM conducted the hyperspectral image collection, preprocessing of images, and developed the pipeline for analysis of images. MM performed hydroponic screening assays and biomass measurements, and SK helped oversee the experimental design of screening. CY and PM provided technical assistance in analysis of images. AM wrote the paper and MM co-wrote the biology parts of the paper. CY, PM, and SK edited the paper.

### Conflict of interest statement

The authors declare that the research was conducted in the absence of any commercial or financial relationships that could be construed as a potential conflict of interest.
