# Supplementary material for: A Novel Approach to Assess Salt Stress Tolerance in Wheat Using Hyperspectral Imaging
Source: Front Plant Sci. 2018 Aug 24;9:1182. doi: 10.3389/fpls.2018.01182 (PMC6117507; doi:10.3389/fpls.2018.01182)
Supplement: Supplementary file 1 [file Table_1.docx]

Supplementary Material

A novel approach to assess salt stress tolerance in wheat using hyperspectral imaging

Ali Moghimi^1*^, Ce Yang^1^, Marisa E. Miller ^2, 3^, Shahryar Kianian^2, 3^, Peter M. Marchetto^1^

^1^ Department of Bioproducts and Biosystems Engineering, University of Minnesota, MN, USA

^2^ Cereal Disease Laboratory, USDA-ARS, MN, USA

^3^ Department of Plant Pathology, University of Minnesota, MN, USA

*** Correspondence:**Ali Moghimi

[moghi005@umn.edu](mailto:moghi005@umn.edu)

**Supplemental Table 1.** Analysis of Deviance for Conventional Biomass Measurements

| **Analysis of Deviance Table for Aerial Biomass** (Type II tests) | | | |
| --- | --- | --- | --- |
|  | **Chisq** | **Df** | **Pr(>Chisq)** |
| Salt Level | 27.31 | 1 | 1.733e-07 *** |
| Genotype | 48.511 | 3 | 1.657e-10 *** |
| Salt Level:Genotype | 12.635 | 3 | 0.005497 ** |

| **Analysis of Deviance Table for Root Biomass** (Type II tests) | | | |
| --- | --- | --- | --- |
|  | **Chisq** | **Df** | **Pr(>Chisq)** |
| Salt Level | 23.9625 | 1 | 9.823e-07 *** |
| Genotype | 41.9167 | 2 | 4.179e-09 *** |
| Salt Level:Genotype | 7.6443 | 2 | 0.05396 . |
| Signif. codes: 0 ‘***’ 0.001 ‘**’ 0.01 ‘*’ 0.05 ‘.’ | | |  |
