# Supplementary material for: A Novel Approach to Assess Salt Stress Tolerance in Wheat Using Hyperspectral Imaging
Source: Front Plant Sci. 2018 Aug 24;9:1182. doi: 10.3389/fpls.2018.01182 (PMC6117507; doi:10.3389/fpls.2018.01182)
Supplement: Supplementary file 2 [file Table_2.docx]

Supplementary Material

A novel approach to assess salt stress tolerance in wheat using hyperspectral imaging

Ali Moghimi^1*^, Ce Yang^1^, Marisa E. Miller ^2, 3^, Shahryar Kianian^2, 3^, Peter M. Marchetto^1^

^1^ Department of Bioproducts and Biosystems Engineering, University of Minnesota, MN, USA

^2^ Cereal Disease Laboratory, USDA-ARS, MN, USA

^3^ Department of Plant Pathology, University of Minnesota, MN, USA

*** Correspondence:**Ali Moghimi

[moghi005@umn.edu](mailto:moghi005@umn.edu)

**Supplemental Table 2.** Linear Mixed Effects Modeling for Conventional Biomass Measurements

| **Linear Mixed Effects Modeling of Aerial Biomass*** | | | | | |
| --- | --- | --- | --- | --- | --- |
| Akaike Information Criterion (AIC) | 188.6683 | | | | |
| Bayesian Information Criterion (BIC) | 213.0949 | | | | |
| Log-Likelihood | -84.33417 | | | | |
|  | Value | Standard Error | DF | t-value | p-value |
| Intercept^1^ | 2.205 | 0.15205739 | 81 | 14.501104 | 0 |
| salt_level200^2^ | -0.0052375 | 0.00107521 | 4 | -4.87115 | **0.0082** |
| co(CS)^3^ | -1.1057504 | 0.18508003 | 81 | -5.974445 | **0** |
| sp(CS)^3^ | 0.0833572 | 0.18508003 | 81 | 0.450385 | 0.6536 |
| Kharchia^3^ | -0.0408333 | 0.18089641 | 81 | -0.225728 | 0.822 |
| salt_level200:co(CS)^4^ | 0.0041496 | 0.00129401 | 81 | 3.206773 | **0.0019** |
| salt_level200:sp(CS)^4^ | 0.0003749 | 0.00129401 | 81 | 0.289705 | 0.7728 |
| salt_level200:Kharchia^4^ | 0.0009923 | 0.001294 | 81 | 0.766813 | 0.4454 |

| **Linear Mixed Effects Modeling of Root Biomass*** | | | | | |
| --- | --- | --- | --- | --- | --- |
| Akaike Information Criterion (AIC) | 137.2866 | | | | |
| Bayesian Information Criterion (BIC) | 161.7131 | | | | |
| Log-Likelihood | -58.64329 | | | | |
|  | Value | Standard Error | DF | t-value | p-value |
| Intercept^1^ | 1.195 | 0.09669393 | 81 | 12.358583 | 0 |
| salt_level200^2^ | -0.0022625 | 0.00068373 | 4 | -3.309058 | **0.0297** |
| co(CS)^3^ | -0.6513636 | 0.13981919 | 81 | -4.658614 | **0** |
| sp(CS)^3^ | 0.0777273 | 0.13981919 | 81 | 0.555913 | 0.5798 |
| Kharchia^3^ | 0.1641667 | 0.13674587 | 81 | 1.200524 | 0.2334 |
| salt_level200:co(CS)^4^ | 0.002236 | 0.00097787 | 81 | 2.286597 | **0.0248** |
| salt_level200:sp(CS)^4^ | -0.0000511 | 0.00097787 | 81 | -0.052294 | 0.9584 |
| salt_level200:Kharchia^4^ | 0.0000848 | 0.00097787 | 81 | 0.086769 | 0.9311 |

*Both models fit by Residual Maximum Likelihood (REML)

^1^Chinese Spring at 0 mM salt

^2^The change (coefficient) when salt level changes from 0 to 200 mM for Chinese Spring

^3^The change in dry weight from Chinese Spring to the indicated genotype when the salt level is 0

^4^The coefficient changes from Chinese Spring to the indicated genotype when the genotype changes from 0 to 200 mM salt
